# Moving to Personalized Medicine Requires Personalized Health Plans

**DOI:** 10.2196/35798

**Published:** 2022-08-04

**Authors:** Adam Powell, Paul Dolan

**Affiliations:** 1 Payer+Provider Syndicate Newton, MA United States; 2 London School of Economics and Political Science London United Kingdom

**Keywords:** quality-adjusted life years, health insurance, personalized outcomes, patient preferences, cost-effectiveness, managed care

## Abstract

When individuals, families, and employers select health plans in the United States, they are typically only shown the financial structure of the plans and their provider networks. This variation in financial structure can lead patients to have health plans aligned with their financial needs, but not with their underlying nonfinancial preferences. Compounding the challenge is the fact that managed care organizations have historically used a combination of population-level budget impact models, cost-effectiveness analyses, medical necessity criteria, and current medical consensus to make coverage decisions. This approach to creating and presenting health plan options does not consider heterogeneity in patient and family preferences and values, as it treats populations as uniform. Similarly, it does not consider that there are some situations in which patients are price-insensitive. We seek to highlight the challenges posed by presenting health plans to patients in strictly financial terms, and to call for more consideration of nonfinancial patient preferences in the health plan design and selection process.

## Introduction

In the United States, there are many health plan designs in use. When individuals, families, and employers select plans, they are typically only shown the financial structure of the plans and their provider networks. This variation in financial structure can lead patients to have health plans aligned with their financial needs, but not with their underlying preferences—such as their desire for their health plan to cover or not cover family planning services. Compounding the challenge is the fact that managed care organizations have historically used a combination of population-level budget impact models, cost-effectiveness analyses, medical necessity criteria, and current medical consensus to make coverage decisions. This approach to creating and presenting health plan options does not consider heterogeneity in patient and family preferences and values, as it treats populations as uniform. Similarly, it does not consider that there are some situations in which patients are price-insensitive. [1] We seek to highlight the challenges posed by presenting health plans to patients in strictly financial terms, and to call for more consideration of nonfinancial patient preferences in the health plan design and selection process.

## Personalization and the Patient

Patients and their families can be directly involved in the process of valuing health plan attributes. Currently, there are several health plan decision support tools, such as Picwell and PLANselect, which help patients and families select health plans by answering questions about their financial preferences. The decision-making process is centered around answering questions related to premiums, deductibles, and other financial characteristics. Clinical questions typically relate to the anticipated frequency of health care utilization and prescription medications used. Once these questions are answered, a number of health plans are presented as options for patients and families, along with information about their financial characteristics (monthly premium costs, copays, and deductibles) and health care providers available in each plan’s network. Given the information provided, it is not possible for patients and families to understand how the coverage they are being offered aligns with their nonfinancial preferences and values. Information to consumers tends to lack transparency and details on the coverage of services for which preferences can vary owing to attitudinal differences, such as complementary and alternative medicine, medical abortion, or care at the end of life.

While current decision support tools simplify the health plan selection process for patients and their families, they ignore the underlying differences in the coverage policies between plans and may match an individual or family with a health plan whose coverage policies are not aligned with their values. The potential for misalignment between health plan coverage and personal values has been highlighted in the United States in the context of abortion, where in 2018, approximately half of US adults surveyed were found to support health plan coverage for abortion and approximately half did not support health plan coverage for it [2]. At present, it is so difficult to fully comprehend health plan details that even human resources departments, who typically make decisions regarding health plan benefit designs on behalf of a company or organization, are challenged. In one instance, the Catholic University of America inadvertently offered a health plan with limited abortion coverage before later discovering that their insurer had modified the plan’s design without informing them [3]. Health plan decision support tools currently do not provide patients and families adequate support in assuring that the coverage policies of the health plan that they are selecting aligns with their beliefs, preferences, and values.

Today, a variety of methods, including standard gambles, time trade-offs, discrete choice conjoint analysis, and willingness to pay are used to elicit public preferences for health care services, with conclusions extrapolated to large and varied populations [4]. Going forward, a more tailored approach could be used in which patients and their families can be directly involved in the process of valuing financial and nonfinancial health plan attributes, and then paired with plans that align with their preferences individually rather than plans reflecting general societal norms that are only tailored on the basis of financial preferences. Moving toward a system in which patient and family preferences are better reflected in plan designs requires a redefining of the plan “shopping” experience. Rather than merely asking consumers whether they would wish to have a higher premium or a higher deductible, or whether they wish to have reduced premiums in exchange for reduced provider choice, health plans can additionally compete on the degree to which preferences over nonfinancial aspects of coverage (eg, the range and duration of health services covered and the provision of nonmedical services addressing the social determinants of health) are being satisfied.

The approach used to determine the services covered by a health plan may need to differ in an environment in which health plans are selected by individuals and employers (as is the case in the United States), rather than by a public payer (as is the case in the United Kingdom). Using quality-adjusted life years (QALYs) to facilitate decision-making related to health plan coverage—as a special task force of the Professional Society for Health Economics and Outcomes Research has recommended that US payers do—may not be fit for purpose if the health outcomes considered in calculating the QALYs generated by an intervention are weighted uniformly for everyone living in the country [5]. The preferences of individuals and employers can vary greatly [6]. Research has also shown that the social value of an incremental QALY is not universal across individuals but instead depends on whether a person is nearing the end of life and may also depend on the person’s prospective burden of illness [7].

Personalized cost-effectiveness analyses may be particularly valuable to people living with disabilities. While individual underwriting was banned in the United States by the Affordable Care Act, personalized cost-effectiveness analyses differ from underwriting in that they can be used to determine the benefits covered by the policy itself, rather than its pricing. Managed care organizations can—and do—offer a range of different health plans, at different pricing, with different attributes. The United States National Council on Disability has called for a moratorium on the use of QALYs in decision-making for Medicare and Medicaid (public health insurance programs) on the grounds that QALYs devalue interventions that extend the lives of people with disabilities and that mitigate the impact of disability on health [8]. Compared to interventions provided to people without disabilities, those provided to people with disabilities generate fewer future QALYs, thus driving discriminatory policies that may deprioritize people with disabilities [9]. This discrimination is exacerbated by the general practice of having people without disabilities participate in the assignment of QALYs, as people without a particular disability rate their expected quality of life with the disability as lower than do people living with that disability [10]. By engaging people with disabilities in the process of designing policies for similarly situated individuals by proactively seeking information on their preferences, plans can develop benefits that are better aligned with the people they serve.

## Personalization and the Health Plan

As we approach an era of personalized medicine, we may need to enter an era of personalized health plans, in which patients and employers can choose from among a wider variety of health plans that differ in both their financial structures and the values that they capture. These include, for instance, the following:

- Should a health plan seek to extend life at all costs?
- Should a health plan offer coverage for services that may shorten or end life?
- Should a health plan cover services that prioritize convenience over quality?
- Should a health plan allow patients the flexibility to choose their provider even if their preferences may lead them to seek high-cost or low-quality providers?
- Should a health plan offer coverage for alternative therapies backed by minimal scientific evidence?

There is no single correct answer to these questions, but in a system in which there is a degree of consumer and employer choice, people and employers can potentially be paired with preference-aligned plans.

Conducting single cost-effectiveness analyses for interventions does not enable payers to adequately deal with the diversity of the patients who they serve. QALYs seek to express the value of changes in quality and length of life in a single metric, and have become a widely used measure of health benefits in cost-effectiveness evaluations [11]. Wrapped up in the logic of the QALY is the premise that a payer will be willing to cover “cost-effective” therapies, and that the QALY gains from a given intervention are assumed to be of the same value, irrespective of the preferences and nonhealth characteristics of the patients [12]. We already recognize, however, that there are some differences among populations, as countries have assigned different weightings to the health outcomes used to compute a QALY, as well as different implied monetary values to a QALY, and thus an intervention with the same costs could be considered cost-effective in one jurisdiction but not in another [13]. Similarly, different individuals have different preferences, and thus there is scope for greater patient participation in determining what is cost-effective for each group or individual rather than simply for society as a whole.

The growth of personalized medicine also raises challenges to the generalizability of QALYs [14]. While a particular small-molecule treatment may be used to address multiple indications, all patients are ultimately taking the same drug. In contrast, when patients access and use a given digital therapeutic for different indications, they may be receiving different interventions, which aim to address different health concerns. For instance, a single app may offer a fully self-guided treatment to people with mild depression but a more expensive, therapist-guided intervention to people with moderate depression [15]. Although the specific app itself is the same in both cases, the treatment it provides and the cost of delivering that treatment varies in accordance with the indication for which it is used. Likewise, the number of QALYs generated by the app vary in accordance with how it is used. Generalizability issues are likely to extend into other forms of treatment as well because personalized medicine increasingly leads to the tailoring of biological and chemical interventions, in place of the traditional “off-the-shelf” treatments used previously.

The standard QALY approach is based on eliciting of the preferences of members of the public over different health outcomes, where the strength of preference is determined by trade-offs against life expectancy or risk of death. There are serious problems with the ability of such preferences to serve as good guides to the relative impact of different health outcomes on peoples’ lives [16]. A more robust and reliable approach might therefore be to conduct assessments that allow peoples’ reports of their well-being and values to be used to determine the relative weights allocated to different health outcomes [17]. By developing a menu of different health plan offerings with different weights, payers can enable patients, families, and employers to more readily select plans that fit their preferences and values, with the understanding that not everyone would assign the same weights to outcomes when determining the QALYs experienced in a health state.

## Conclusions

Irrespective of the details of valuing the outcomes produced by medical interventions, more patient participation in determining health plan coverage decisions will become necessary, especially as personalized medicine is playing an ever-increasing role in care. For more accurate assessments of the utility produced by interventions covered by health plans to be made, patients must more actively share their own preferences so that they may be properly accounted for in the decision-making model. Payers should work with patients and employers toward developing health plan population–specific coverage decisions more consistent with the preferences of the populations that these plans serve. Ultimately, personalizing medicine will require a new, more tailored approach to determine the health services that health plans should cover.

## Abbreviations

QALY: quality-adjusted life year
